# Quality of care: not hospital but operator volume of pacemaker implantations counts

**DOI:** 10.1007/s12471-013-0506-8

**Published:** 2013-12-18

**Authors:** N. M. van Hemel

**Affiliations:** Utrecht University, UMC Utrecht, Utrecht, Netherlands

**Keywords:** Pacemaker implantation, Operator experience, Hospital volume, Quality of care

## Abstract

Literature about pacemaker (PM) implantations shows that several clinical and technical factors determine the short- and long-term complications after the intervention. Annual hospital volume, however, does not negatively affect complications in contrast with the cumulative experience of the operator. In view of this observation, the current required number of 20 to 30 first PM implantations for cardiology training does not match standards for quality of care. In addition, concentration of implants and replacement of pacemakers to a limited number of operators per hospital to comply with the increasing demands of patients and other parties has to be seriously considered.

Since 1959, pacemaker (PM) implantation exclusively for bradycardia indications has become a routine procedure. Numerous measures have been undertaken to ameliorate the surgical procedure as well as the technical performance of the implanted device. In the very early days epicardial leads were only available requiring thoracotomy and therefore at that time implantations were carried out in the operating theatre by cardiac surgeons. However, when endocardial leads became available, cardiologists took over the implantation procedure and the catheterisation room, with its gradually improving imaging techniques, replaced the operating theatre. Early studies could not show any difference in complication rates of first PM implantation, specifically regarding infection, between the two facilities [1], and the costs of implantation in the catheterisation room were clearly lower [2].

However, these observations should be envisaged with caution due to the small sample size, retrospective study design and generally short follow-up after first PM implantation. Focussing on the incidence of infection after first PM implantation, the low prevalence (varying from 0.6 to 3 %, [3–5] requires a considerable number of patients for a comparative study to obtain a clear answer on the best preventive measures [6]. PM replacement, for whatever reason, is associated with a remarkably higher complication rate [7], in particular infection [8]. Cardiac device infections are serious, sometimes life-threatening [9] and cause high additional hospital costs [10], specifically due to the intensive and long-term care. It is emphasised that a study of the outcome of PM replacements comparing this intervention in the catheterisation room versus the operating theatre, has never been published.

Nevertheless, a first PM implantation or replacement of the electronic generator for whatever reason is today thought to be an easy, safe and 1-day procedure that can be done in the catheterisation room of all cardiology departments of the Netherlands. This applies for single-chamber versus double-chamber PM implantations [3]. Antibiotic prophylaxis plus antiseptics within 1 h before the procedure greatly reduces surgical infections [5, 6] and this measure contributes strongly to the opinion about the ease of PM implantation or replacement. It should be emphasised that PM treatment is associated with a clearly lower complication rate and less adverse impact on patient outcome [11] than ICD implantation with or without a cardiac resynchronisation device, which requires a special licence in our country,

Assessment of quality of care encompasses established matters as the appropriateness, safety and cost-effectiveness of a treatment and these components have become important chapters in patient care and patient counselling. Regarding safety of a PM implantation or replacement, reflected by the type and number of complications, one can speculate about the relationship between the annual hospital volume of these interventions and the number of immediate and late complications. The pre-implantation morbidity and technical complexity of PM implantation strongly determine the acute and late complication rates: a reduced left ventricular function, a dilated right ventricle, heart failure and infection at the time of first implantation, are predictors for an unfavourable outcome [12]. Hospital volume is often thought to be of predictive value but the Followpace study [4] could not observe any relationship between early and late complication rate with the annual hospital volume of 23 Dutch general and university hospitals. Several other device studies underlined this outcome [13, 14] and therefore hospital volume as such cannot be considered an unfavourable determinant for complications.

Operator experience, defined as the cumulative number of PM implantations or annual implantations per operator, appears more related to complications than the annual hospital volume. Tobin et al. [15] indeed noted an inverse relationship between the yearly number of cases per operator and complication rate (*r* =−0.90, *p* = 0.002) and experience in terms of years of PM implantation (*r* = 0.81, *p* = 0016): operators doing >40 cases/year and/or >10 years of experience had the lowest complication rates. Wiegand et al. [16] confirmed this observation within a study of anticoagulation or antiplatelet management in patients who underwent PM or ICD implantation. Implanter experience was an independent predictor for pocket bleeding as well as for the need for reoperation for this complication: HR 0.61 for operators with >100 implantations experience and HR 1.75 for those <50 ones. Eberhardt et al. [12] defined three levels of cumulative experience, namely <50, 50–100 and >100 PM implantations and observed obviously lower hazard ratios for operation and fluoroscopic time and number of operative revisions in the group of operators with largest experience. Furthermore, this study showed that the complication rate of single- and dual-chamber PM implantations was similar in experienced hands in contrast to inexperienced operators. The authors calculated a relative risk reduction for complications of 0.6 % for each implanted PM and concluded that ‘more than 100 PM implantations seem to have been necessary to achieve a low complication rate’.

These data indicate that operator experience of PM implantations clearly counts to promote quality of care. When cardiology training in the Netherlands is considered (see paragraph 8.4.2010), a minimum of 20 or 30 first PM implantations for competence level 1 and 2, respectively, is prescribed (April 2010: see www.nvvc.nl/opleiding). These numbers do not suffice at all to comply with the above-mentioned observations of the necessity of a large operator experience [14, 17]. Furthermore, the April 2013 standards of the Netherlands Rhythm Association and the Committee for Quality of Care of the Netherlands Society of Cardiology require annually 50 PM implantations per hospital and 25 per operator (see www.nvvc.nl/richtlijnen/indicatoren-en-normen). That includes one PM implantation in the hospital per week or two monthly implantations per operator. One can seriously doubt whether these numbers will fulfil the requirements of today’s proposed high(er) levels of care.

Concentration of PM implantations to a small number of cardiology trainees appears unavoidable and requires adaptation of the training curriculum to cope with surgical techniques for device pocket, lead insertion and PM technology, which is now carried out annually in over 10,000 Dutch patients with a too slow heart rhythm. This point of view should be extended to licensed cardiologists by assigning ‘hot shots’ who perform at least 50 first PM implantations per year. This also holds for the implantation of resynchronisation devices without ICD. This new scenario of standards will undoubtedly promote better PM treatment in a time of increasing comorbidity and frail and very old patients [18] who are better informed than ever before. In addition, less complications facilitate the nationwide application of remote follow-up of PMs, which is unavoidable in the coming years of budget constraints [19].
